# The contribution of the smartphone use to reducing depressive symptoms of Chinese older adults: The mediating effect of social participation

**DOI:** 10.3389/fnagi.2023.1132871

**Published:** 2023-04-06

**Authors:** Rong Ji, Wei-chao Chen, Meng-jun Ding

**Affiliations:** ^1^School of Journalism and Communication, Hunan Normal University, Changsha, China; ^2^School of Literature and Journalism, Xiangtan University, Xiangtan, China

**Keywords:** elderly, smartphone use, depression symptoms, influence mechanism, political participation, voluntary participation, active leisure participation, passive leisure participation

## Abstract

**Background:**

Depression is a prevalent mental health disorder. Although Internet use has been associated with depression, there is limited data on the association between smartphone use and depressive symptoms. The present study aimed to investigate the relationship between smartphone use and depressive symptoms among older individuals in China.

**Methods:**

5,244 Chinese older individuals over the age of 60 were selected as the sample from the China Longitudinal Aging Social Survey (CLASS) 2018 dataset. The dependent variable “depression symptoms” was measured using the 9-item Center for Epidemiologic Studies-Depression (CES-D) scale. The study employed multiple linear regression to investigate the relationship between smartphone use (independent variable) and depressive symptoms in older people. Thorough analyses of robustness, sensitivity, and heterogeneity were conducted to ensure the robustness and sensitivity of the findings. Additionally, mediating effect analysis was performed to elucidate the mechanism through which the dependent and independent variables were related.

**Results:**

Empirical study indicated that smartphone use had a negative impact on depressive symptoms among older adults, specifically leading to a reduction in such symptoms. The above-mentioned result was verified through endogenous and robustness tests. The heterogeneity analysis revealed that older individuals aged 70 years and above, male, and residing in urban areas exhibited a stronger association between smartphone use and depressive symptoms. Furthermore, the mediating effect model indicated that political participation, voluntary participation, and active leisure participation mediated the relationship between smartphone use and lower levels of depression symptoms among the older adults. However, passive leisure participation had a suppressing effect on the relationship between smartphone use and reduced depressive symptoms among the older adults.

**Limitations:**

The causal relationship between variables required further investigation with a longitudinal design.

**Conclusion:**

These findings suggested that smartphone use may be considered an intervention to reduce depression symptoms among older people by increasing levels of political participation, voluntary participation, and active leisure participation.

## 1. Introduction

Aging has emerged as a major global public health concern due to the significant rise in the number of older people. As the country with the largest older population in the world, China’s aging rate has exceeded the world average level (Chen and Chan, 2014). It is estimated that China’s older population over 60 will exceed 358 million by the end of 2030 (Peng and He, 2017), posing huge challenges to the supply of medical and public services. Depression is a prevalent condition and a public health concern (Reuman et al., 2022), there is a growing body of evidence suggesting that depression has a negative impact on the survival of older adults (Zivin et al., 2015).

The continuous advancements in information and communication technologies (ICTs) have enabled older adults to access the Internet and engage in online activities as a part of their daily lives. The smartphone, one of the world’s fastest-diffusing communication technologies, integrates audio, video, and text with a display screen, and combines the characteristics of interpersonal and mass communication. In China, the smartphone has experienced unprecedented growth, with over 950 million individuals owning one in 2021 (Richter, 2022). Among the different types of devices used to access the Internet, the smartphone is the most accessible in the daily lives of seniors. Research has indicated that seniors are increasingly using smartphones to send and receive video/audio calls, download applications, browse the Internet, and participate in various other activities (Neves et al., 2019; Brenner, 2022), which will, in turn, strengthen ties with family and friends, reduce social isolation and loneliness, and improve the quality of their life. Therefore, smartphone use may alleviate the depressive symptoms of older adults (Minagawa and Saito, 2014; Lin et al., 2020). However, little research has focused on the relationship and mechanism between smartphone use and depressive symptoms among the older adults.

According to the existing studies, social participation, which is an integral part of the successful aging strategy (Aw et al., 2017; Douglas et al., 2017), has a positive impact on older adults’ physical and mental health (Alma et al., 2012; Liu et al., 2019). Moreover, the World Health Organization (WHO) proposes social participation as one of the key policy frameworks in response to concerns about the globally urgent aging situation (World Health Organization [WHO], 2015). Is participation in social activities a very important intermediary factor between smartphone use and the depressive symptoms of older adults?

Therefore, this study hypothesizes that there is a relationship between smartphone use and depressive symptoms in older individuals and that social participation serves as a mediator between the two variables. Specifically, the hypothesis posits that augmented smartphone use among the older adults leads to increased social participation, ultimately resulting in a reduction in depressive symptoms.

## 2. Literature review

### 2.1. Effect of smartphone use on depressive symptoms of the older adults

Current research generally agrees that smartphone use can contribute to maintaining the health status of the older population. Specifically, research findings can be summarized into two aspects. Specifically, the research results can be summarized in the following two aspects.

First, it was suggested that technology use had a positive impact on mental health. Grossman’s health theory supported the notion that technology use had a stronger correlation with the mental wellbeing of the older adults. Chopik (2016) found that social technology use (i.e., social networking sites, online chatting/instant messaging, using a smartphone) was associated with fewer depressive symptoms. Kim et al. (2020) reported a positive relationship between smartphone use and life satisfaction among older adults in South Korea. Other studies have also shown that information and communication technology (ICT) is associated with improved mental health and wellbeing (Cotten et al., 2012; Chen and Schulz, 2016). Specifically, the informational use of smartphones can not only enhance health knowledge and promote a healthy lifestyle for the older adults (Boontarig et al., 2012) but also provide access to up-to-date news and resources for regulating affective states and mental health (Hoffner and Lee, 2015).

Second, smartphone use was found to have a statistically significant negative impact on mental health. Some researchers found the misuse of smartphones may have a negative effect on mental health, particularly among adolescents (George and Odgers, 2015). Wang et al. (2018) discovered that using smartphones for entertainment purposes, such as playing games to pass time, may decrease the likelihood of face-to-face interactions and lead to increased feelings of loneliness among the older adults.

One reason for these divergent findings could be differences in populations studied. For instance, certain studies may concentrate on older adults who are already proficient in using smartphones, while others may analyze older adults who are less familiar with technological devices. The former group may be more inclined to reap the benefits of smartphone usage, whereas the latter group may encounter challenges in utilizing technology, resulting in frustration and decreased mental wellbeing.

Another potential factor is the methodological approach used by researchers. For example, studies that rely on self-reported measures of smartphone use may be subject to bias, as older adults may not accurately report their actual usage. Additionally, studies that are cross-sectional in nature may not be able to establish causality between smartphone use and mental health outcomes.

Smartphones offer a range of features and apps that promote physical and mental activity, such as fitness trackers, brain training games, and social media platforms. These activities have been shown to have a positive impact on mood and cognitive function, potentially contributing to a reduction in depressive symptoms among older adults.

Grounded on the above literature, this study proposes the following hypothesis:

H1: Smartphone use is associated with a decrease in depressive symptoms among older adults.

### 2.2. The mediating role of social participation in the relation between smartphone use and depressive symptoms among older adults

Social participation was a very valuable concept in old age, as it was considered one of the most critical components of health and a key component of successful aging among the older adults (Utz et al., 2002). Despite its importance, the concept of social participation received less attention, and there was no widely accepted definition of it (Piškur, 2013). Therefore, various measuring methods were proposed.

According to the international classification of function (ICF), social participation was defined as the activities and obligations required to engage in social life outside the family environment, such as in society, the community, and civil society (Dahan-Oliel et al., 2008). This study categorized social participation into three groups, based on the studies by Kobayashi et al. (2015), this study categorizes social participation into four groups: political participation, voluntary participation, active leisure participation, and passive leisure participation.

#### 2.2.1. The mediating role of political participation in the relation between smartphone use and depressive symptoms among older adults

Voting in local elections was one of the most common political behaviors in rural and urban China (Duckett and Wang, 2013). Earlier research had found that higher ownership of smartphones enabled older people to connect with a larger network of contacts (Hutto et al., 2015), and consequently improved their skills and opportunities for social participation (Quinn, 2018). Ohme (2019) had observed a rather strong relationship between social media use and political participation among the older adults. Empirical findings had suggested that political participation might be positively associated with mental health for European people (Croezen et al., 2015). Based on the literature cited above, this study proposes the following hypothesis:

H2: Political participation mediates the relationship between smartphone use and lower levels of depressive symptoms among older adults.

#### 2.2.2. The mediating role of voluntary participation in the relation between smartphone use and depressive symptoms among older adults

Volunteering meant any activity in which time is given freely to benefit another person, group, or organization (Wilson, 2000). Previous studies had shown that age was an important factor that motivated people to engage in voluntary activities, implying that the older adults were more likely to engage in voluntary activities (Wilson, 2012). For example, some older adults used volunteer work as a substitute for their jobs after retiring (Hank and Stuck, 2008). In the digital age, technological advances heavily influenced how voluntary efforts were organized.

First, smartphone and Internet use could encourage older people to engage in voluntary activities more frequently. Compared to young people, Internet use could strengthen voluntary participation among the older adults (Nie and Erbring, 2002). For instance, Mukherjee’s study had found that older people could overcome coordination problems in volunteer work by using the Internet (Mukherjee, 2011). Moreover, smartphones made it easy for individuals to obtain relevant information about voluntary activities, which in turn encouraged them to participate (Gao, 2020).

Second, engaging in voluntary activities was linked to a reduction in depressive symptoms among the older adults. Activity Theory believed that older people who frequently engage in various activities tended to report higher levels of health (Fernández-Ballesteros et al., 2001). Furthermore, some researchers had found that volunteering is helpful in alleviating depression in the older adults, especially those over 65 years old (Musick and Wilson, 2003). Besides, Li and Ferraro (2005) had found that participation in formal voluntary activities could help reduce depression symptoms. This was mainly because informal volunteer work was less socially acceptable and required a stronger sense of social responsibility than formal volunteer work, which might have offset the positive effects of volunteering on depression.

Based on the literature cited above, this study proposes the following hypothesis:

H3: Voluntary participation mediates the relationship between smartphone use and lower levels of depression symptoms among the older adults.

#### 2.2.3. The mediating role of leisure participation in the relation between smartphone use and depressive symptoms among older adults

Leisure participation was defined as engagement in enjoyable activities during time free from obligations or responsibilities (Pressman et al., 2009). From the perspective of leisure engagement attributes, some scholars divided it into active and passive leisure activity (Holder et al., 2009). Gordon and Caltabiano (1996) classified passive leisure activities as watching television/videos, reading, sitting around feeling bored, listening to music, writing correspondence, relaxing, thinking, doing nothing, and telephone conversations. Active leisure activity often encompassed home maintenance, physical activities or exercise, special events or cultural activities, clubs or organizations, and so on (Cho et al., 2017).

**First**, the smartphone could encourage older people to engage in leisure activities more frequently. First, the older adults could easily obtain relevant information about social activities and community events through the Internet, which in turn stimulated them to participate in offline-related activities (Czaja, 2017). Second, the smartphone became the main tool for the entertainment and online communication of the older adults. Näsi et al. (2012) found that Internet use in old age had a strong positive correlation with the number of different leisure activities amongst Finnish seniors. According to another study (Zhang and Zhu, 2021), smartphone use enriched the entertainment activities and leisure lives of the Chinese older adults.

**Second**, regarding the relationship between leisure participation and the older adult’s depression, the activity theory suggested that consistent engagement in social and healthy leisure activities is more likely to result in better mental health for seniors (Fernández-Ballesteros et al., 2001). Additionally, research has shown that social media communication is associated with increased levels of perceived social support and social contact, which in turn are linked to lower levels of loneliness among older adults (Zhang et al., 2020). Active leisure was negatively associated with depressive symptoms, anxiety, and loneliness (Lampinen et al., 2006). Passive leisure activities such as watching television, listening to the radio, or reading, had been found to have a negative or no effect on life satisfaction levels, quality of life, or mental health (Akbarly et al., 2009). Additionally, passive leisure activities that were enjoyed alone may impede a socially healthy aging process (Chul-Ho et al., 2020).

Based on the literature cited above, this study proposes the following hypotheses:

H4a: Active leisure participation mediates the relationship between smartphone use and lower levels of depression symptoms among the older adults.

H4b: Passive leisure participation passive leisure participation has a noticeable dampening influence on the effect of smartphone use to reduce levels of depressive symptoms.

## 3. Materials and methods

### 3.1. Data sources

We adopt the Data from the China Longitudinal Aging Social Survey (CLASS) for the downstream empirical analysis. CLASS is a national longitudinal social survey organized and implemented by the Renmin University of China, to comprehensively understand the basic situation of the older adults. The respondents of CLASS are Chinese older people over 60. Data of CLASS 2018 has an original sample size of 11,418. With the preprocessing of missing values and outliers, a total of 5,244 valid samples are obtained. Empirical analyses in our study are realized by two different statistical software programs: R (a freely available statistical software, version 4.1.0) and Stata (version 16.0).

### 3.2. Variables and measurements

#### 3.2.1. Dependent variable

In this study, the dependent variable “depressive symptoms” is measured by a subset (9 items) from the Center for Epidemiologic Studies-Depression (CES-D) scale (Radloff, 1977). The 9-item CES-D scale has been broadly used to measure depressive symptoms in older persons and also has been validated for studies of Chinese adults (Wang et al., 2019; Zhou et al., 2020). In the CLASS questionnaire, depressive symptoms are measured by the questionnaire: “How often did you have the following nine feelings in the past week?” The respondents are asked to report the frequency of feelings positive feelings (feeling happy, enjoying life, feeling pleasure), negative feelings (feeling lonely, feeling upset), marginalization feelings (feeling useless, having nothing to do), and somatic symptoms (having a poor appetite, having trouble sleeping) (Tang et al., 2019). For each activity (item), the respondents’ answers are coded as 1 = Never, 2 = Sometimes, 3 = Often. The positive items are recoded, and the average score of above 9 items is calculated to measure the level of depressive symptoms of the older adults. A higher score indicated a higher level of depressive symptoms (Cronbach’s α = 0.706). Depression ranges from 9 to 27 with a mean of 15.766 and a standardized deviation of 3.083.

#### 3.2.2. Independent variable

Referring to the study by Wang (2020), smartphone use is measured by a single-item question “Are you using a smartphone now?.” It is a binary variable with 1 indicating smartphone use and 0 for others.

#### 3.2.3. Mediating variables

Previous studies have suggested that there is a significant correlation between smartphone use and social participation (Kim and Kim, 2021), and social participation might have a contribution to reducing levels of depressive symptoms (Croezen et al., 2015). Based on this evidence, we select social participation (e.g., political participation, voluntary participation, active leisure participation, and passive leisure participation) as mediating variables.

To measure political participation, referring to the study by Kong (2021), we use the following question in the CLASS questionnaire: “Have you participated in the voting of the residents’ committee or villagers’ committee in the past 3 years?.” The respondents’ answers are coded as 0 = No, 1 = Yes.

To measure voluntary participation, referring to the study by Chai and Guo (2020), we use the question: In the past year, have you gotten involved in the following seven volunteer activities? (1) Community security patrols; (2) Caring for other elderly/children; (3) Environmental sanitation protection; (4) Mediating neighborhood disputes; (5) Accompanying chat; (6) Volunteering services requiring professional skills; (7) Caring and educating the next generation. For each activity, the answers are coded as 1 = Yes and 0 = No. If older adults participated in any of these activities, they were assigned a score of 1; they were assigned 0 if they did not participate in any voluntary activities. Leisure participation is constructed from the question “In the past year, have you engaged in the following activities (excluding those who participate through the Internet).” Referring to the study by Chul-Ho et al. (2020), active leisure participation includes: (1) “religious activities,” (2) “attending senior college or training courses,” (3) “playing mahjong, chess, cards, etc.,” and (4) “Public Square Dance (Guang Chang Wu).” Passive leisure participation includes watching TV, listening to the radio, reading books, reading newspapers, and so on. For each activity (item), the options are 0 (No) and 1 (Yes). We created a dummy variable coded 1 if the respondent reported participation in any of these leisure activities and 0 if they did not.

#### 3.2.4. Control variables

Referring to Wang et al. (2019) and Liu et al. (2022), the control variables selected in this study mainly include gender (male, female), age, marital status (married, widowed/divorced/unmarried), household (rural, urban), education (uneducated, primary school, secondary school, and high school and above), work status (retired, not retired), housing situation (not owning a house, owning 1 house, owning 2 houses and above), family income, number of children, ADL score (the activities of daily living), IADL score (the instrumental activities of daily living), number of comorbid chronic disease, and living region (eastern, central, and western).

The CLASS survey contains information on eleven ADLs: making phone calls, grooming, dressing, bathing, feeding, taking medication, controlling urination, controlling defecation, going to the toilet, transferring from bed to chair, and indoor mobility. All of the ADL questions are measured on a three-point scale: 0 = I cannot do it, 1 = I need help, and 2 = I do not need any help. Adding up the scores for each ADL question, we constructed an ADL disability variable. The values of this variable range from 2 (severe ADL disability) to 22 (no ADL disability). The Cronbach’s alpha coefficient of this variable is 0.85, which indicates excellent scale reliability. The survey contains information on seven IADLs including going up and down the stairs, having a fall, walking outside, traveling on transportation, shopping, managing money, lifting something that weighs 10 pounds, cooking, and doing housework. The IADL questions were measured on the same three-point scale. Adding up the scores for each question, the IADL disability variable has a value ranging from 2 (severe IADL disability) to 18 (no IADL disability). The Cronbach’s alpha coefficient for this variable is 0.81. The physical disability status is constructed from the question “whether you had one of the following health problems (covered 23 chronic diseases),” including hypertension, diabetes mellitus, arthritis, cerebrovascular disease, liver disease, and so on. The number of comorbid chronic disease were further categorized into “no chronic illness” = 0, “have one chronic condition” = 1, and “have two and more chronic conditions” = 2.

The living region (Eastern, Central, and Western) is represented by two dummy variables. The descriptive statistical analysis of all variables is shown in Table 1.

**TABLE 1:** Descriptive statistics of variables (*n* = 5,244).

Variable name	Mean value/%	Variable name	Mean value/%
Depression symptoms	15.744	Work status (Not retired) (%)	24.9
Smartphone use (%)	28.5	Family income (Thousand Chinese Yuan per year)	9.45
Social participation (%)		Number of children	2.49
–Political participation	41.6	Housing ownership (%)	
–Voluntary participation	16.9	–Not owning a house	4.0
–Active leisure participation	40.0	–Owning 1 house	88.2
–Passive leisure participation	80.3	–Owning 2 houses and above	7.8
Gender (Male) (%)	50.3	ADL score	21.59
Age	70.73	IADL score	17.29
Household (%)		Number of comorbid chronic disease (%)	1.76
–Rural	47.3	–0	20.0
–Urban	52.7	–1	31.5
Education (%)		≥2	48.5
–Uneducated	26.7	Living region (%)	
–Primary and secondary school	61.8	–Eastern	39.0
– High school and above	11.5	–Central	36.3
Marital status (Married) (%)	71.5	–Western	24.7

### 3.3. Empirical model

Since the dependent variable is continuous, linear regression is adopted to analyze the effect and influence mechanism of smartphone use on depressive symptoms. Denote *X* = (*X*_1_,⋯, *X*_14_) as all 14 control variables (including dummy variables), *smartphone* as smartphone use, and *Depression* as the dependent variable. For the dependent variable, we construct the following multiple linear regression


(1) $$D\,e\,p\,r\,e\,s\,s\,i\,o\,n_{i}=\alpha +\beta \,s\,m\,a\,r\,t\,p\,h\,o\,n\,e_{i}+\gamma \,X_{i}+\varepsilon_{i}$$

Where *i* denotes the ith individual, *Depression*_*i*_ represents its depressive symptoms level, *smartphone*_*i*_ represents smartphone use, α is the intercept term, and β and γ = (γ_1_, ⋯, γ_14_)^*T*^ represent the regression coefficients for the independent and control variables, respectively. ε_*i*_ is the error term.

We also conduct robustness analysis by adopting the propensity score matching (PSM) method. Based on the independent variable of smartphone use, the samples can be divided into the treatment group (smartphone users) and the control group (non-smartphone users). We then identify control variables as many as possible that affect both the dependent variable depressive symptoms and the independent variable smartphone use. The treatment effect of smartphone use on the depressive symptoms of older adults is as follows:


(2) $$Prob\left(D_{i}=1|X_{i}\right)=\frac{e^{\alpha +\sum_{j=1}^{14}\gamma_{j}\,X_{i\,j}}}{1+e^{\alpha +\sum_{j=1}^{14}\gamma_{j}\,X_{i\,j}}}$$

In Equation (2), *Z*_*i*_ is the control variable of the *i*th older adult. *D*_*i*_ is the indicator variable. *D*_*i*_ = 1 indicates that the *i*th older adult has a smartphone, and *D_i_* = 0 indicates that older adults *i* do not have a smartphone.

To further obtain robust matching results, this study used three common matching algorithms, including K-nearest neighbor matching, radius matching, and kernel matching. The average treatment effect (ATT) for depressive symptoms is given as


(3) $$ATT=E\left(Depression_{i}^{T}-Depression_{i}^{C}/D_{i}=1\right)$$

$$=E\left(Depression_{i}^{T}/D_{i}=1\right)-E\left(Depression_{i}^{C}/D_{i}=1\right)$$

In Equation (3), $Depression_{i}^{T}\text{and}Depression_{i}^{C}$ represent the depressive symptoms of the treatment and control groups, respectively.

## 4. Results

### 4.1. Descriptive analysis

Table 1 presents the descriptive statistics of all variables. The results indicate that the overall level of depressive symptoms among the older adults is relatively high, with a mean of 1.36 and a maximum value of 1.87. Moreover, only 26.4% of the older participants use a smartphone. The proportions of older adults participating in political activity, voluntary activity, and leisure activity are 41.2, 16.1, and 82.3%, respectively.

The average age of the samples is 70.7378, 50.3% are male. Furthermore, 71.5% of the participants are married, and 52.7% of them reside in rural areas. A majority of the participants (61.8%, *n* = 3,242) have a primary or secondary school education. The proportion of non-retired participants is 24.9%. Most of the participants own 1 house (*n* = 4,626, 88.2%). In terms of geographic distribution, 39.0% of the older participants live in the eastern region, while 36.3% live in the central region.

### 4.2. Benchmark regression

We first conducted a multicollinearity test and found that the variance inflation factor (VIF) was well below the critical value of 10, with a mean of 1.59 and a maximum value of 2.19. Therefore, we concluded that multicollinearity did not exist in our data. Next, we used linear regression to analyze the data further. Based on the least squares estimation, the results are presented in Table 2.

**TABLE 2:** Linear regression of smartphone use on depression symptoms.

Variables	Model 1	Model 2
Smartphone use	−1.821*** (0.090)	−0.816*** (0.103)
Gender		0.128 (0.081)
Age		0.024*** (0.007)
Marital status		−0.362*** (0.094)
Household		0.592*** (0.111)
Education		−0.345*** (0.074)
Work status		−0.151 (0.104)
Housing ownership		−0.726*** (0.119)
Family income		−0.272*** (0.041)
Number of children		−0.119*** (0.036)
ADL score		−0.093** (0.036)
IADL score		−0.106*** (0.028)
Number of comorbid chronic disease		0.475*** (0.051)
Eastern		−1.372*** (0.114)
Central		−0.105 (0.103)
*N*	5244	5244
*R* ^2^	0.072	0.174
*F* statistic	410.738***	79.322***

*p < 0.1, **p < 0.05, ***p < 0.01. The parentheses are standard errors.

As shown in Table 2, a significant negative correlation has been observed between smartphone usage and depressive symptoms in older adults, indicating lower levels of depression; thus, H1 was accepted. When examining the control variables in Model 2, we found that older adults who were married, had higher levels of education, owned more houses, had higher family income, had more children, and scored higher on both ADL and IADL measures had lower levels of depressive symptoms than their counterparts. Conversely, older adults who were older in age, lived in urban areas, and had more chronic diseases had higher levels of depression. In terms of geographic location, those living in the eastern regions exhibited lower levels of depressive symptoms than those living in the western region. However, gender and work status had no significant effect on depressive symptoms.

### 4.3. Results of robustness analysis

To obtain the net effect of smartphone use on depressive symptoms in older adults, the propensity score matching (PSM) method is adopted to test the robustness of the linear regression results. We first divide the sample into two groups: the treatment group (using the smartphone) and the control group (not using the smartphone). Before applying PSM, we conduct a balance test to ensure that there are no systematic differences between the two groups in terms of the independent variables after matching. We then use PSM to generate a matched comparison group for our analysis. This helps to mitigate the potential biases that may arise from non-random assignment of smartphone use and control groups.

The results of the balance tests based on radius matching are presented in Supplementary Table 1. We can obtain that the standardized deviation of all variables is controlled within the desired 5% after matching. All of the *t*-values are not significant after matching, which shows that after applying the PSM, the difference between the treatment and control groups is not significant, indicating a better matching effect. The results show that the method of PSM is similar to the results of random experiments, indicating a better matching effect.

This paper firstly estimates ATT both before and after matching. As shown in Supplementary Table 2, the ATT before matching is significantly higher than the ATT after matching. This suggests that if the selection bias is not considered, the influence of smartphone use on the depressive symptoms will be overestimated. Three different matching methods are employed to calculate the ATT, and the results are consistent across all methods, indicating the robustness of the findings. In conclusion, the study provides evidence that smartphone use is associated with a reduction in depressive symptoms among the older adults.

### 4.4. Sensitivity analysis

We also conduct a sensitivity analysis to further test the robustness of the results. We use the command “rbounds” in STATA to conduct the “Rosenbaum bounds” analysis, which is commonly used in the existing studies for sensitivity analysis. In Rosenbaum’s approach, the sensitivity analysis aims to ensure that no significant hidden biases exist, and Γ (gamma) is an important index that can indicate the level of sensitivity of the study. Γ = 2 is a generally used threshold to claim that the study is free of hidden bias (Lin et al., 1998).

The results of the sensitivity analysis are displayed in Supplementary Table 3. Ã ranges from 1 to 2.6. When Ã increases to 2.4 (larger than the threshold 2), we can observe a significant sensitivity. Therefore, the sensitivity analysis shows that the results of Table 2 are robust, which can partly support that the PSM analysis results are reliable.

### 4.5. Results of heterogeneity analysis

The effect of smartphone use on the depressive symptoms of the older adults may present heterogeneity among different subgroups. Therefore, this study conducts a heterogeneity analysis based on control variables age, gender, and living region.

The results of the heterogeneity analysis are shown in Table 3. We can see that between the two subgroups of different ages, the effects of smartphone use on the depressive symptoms present heterogeneity. Specifically, smartphone use has a significant negative effect on the depressive symptoms among the older adults aged 60 to 69, but not so much for the adults over 70. Between the two subgroups based on gender, the smartphone presents homogeneous (significant) effects on depression symptoms. Additionally, the prevalence of depressive symptoms among older adults appears to be lower in rural areas compared to their urban counterparts when utilizing smartphone.

**TABLE 3:** Heterogeneity analysis of the impact of smartphone use on depression symptoms.

Variables	Age		Gender		Household	
	**60–69**	**≥70**	**Female**	**Male**	**Rural**	**Urban**
Smartphone use	−1.017*** (0.133)	−0.313** (0.172)	−0.934*** (0.147)	−0.685*** (0.144)	−1.087*** (0.182)	−0.657*** (0.129)
Control variables	Yes	Yes	Yes	Yes	Yes	Yes
*N*	2738	2506	2604	2640	2481	2763
*R* ^2^	0.197	0.110	0.195	0.157	0.150	0.183
*F* statistic	49.15***	23.30***	46.20***	36.23***	32.34***	45.32***

*p < 0.1, **p < 0.05, ***p < 0.01. The parentheses are standard errors.

### 4.6. Mechanism analysis

The results of the linear regression discussed above also suggest smartphone use is negatively associated with lower levels of depression in older adults. Here, we further explored whether social participation is an intermediary factor driving this relationship. The single-step multiple mediation analysis introduced by Hayes (2009) with bootstrapping using 2,000 bootstrap samples and a 95% Bias Corrected (BC) bootstrap confidence interval (CI) is used in this study.

As shown in Table 4, the results indicate that political participation, voluntary participation, and active leisure participation all play a mediating role in the relationship between smartphone use and the depression symptoms, with indirect effects −0.018, −0.088, and −0.050, and 95% CI [−0.037, −0.005], [−0.131, −0.055], and [−0.093, −0.009], respectively; thus, H2, H3, and H4a was accepted. Hence, the indirect effect of smartphone use to reduce levels of depressive symptoms is mediated through political participation, voluntary participation, and leisure participation. Previous studies unveiled that smartphone use and mobile Internet access have a positive effect on political participation, voluntary participation, and active leisure participation (Boulianne, 2017; Wang et al., 2020; Dou, 2021). Meanwhile, political participation, voluntary participation, and active leisure participation have a negative impact on depression symptoms among the older adults, which is consistent with prior studies (Van Willigen, 2000; Lin et al., 2019; Jeong and Park, 2020).

**TABLE 4:** Estimation results of mediating effects.

Hypothesis	Path	Effect	Boot SE	CI = 95%		Significance	Decision
				**LLCI**	**ULCI**		
	Direct effect	−0.696	0.107	−0.925	0.498	Significant	
	TOTAL	−0.816	0.105	−1.033	−0.626	Significant	
	Indirect effect						
H2	(1) SU → PP→ DS	−0.018	0.008	−0.037	−0.005	Significant	Accepted
H3	(2) SU → VP → DS	−0.088	0.019	−0.131	−0.055	Significant	Accepted
H4a	(3) SU → ALP → DS	−0.050	0.021	−0.093	−0.009	Significant	Accepted
H4b	(4) SU → PLP → DS	0.036	0.012	0.014	0.061	Significant	Accepted

Boot SE, bootstrap standard error; LLCI, lower limit confidence interval; ULCI, upper limit confidence interval; SU, smartphone use; PP, political participation; VP, voluntary participation; ALP, active leisure participation; PLP, passive leisure participation; DS, depressive symptoms.

As for the passive leisure participation, 95% CI [0.014, 0.061] does not contain zero. Thus, the direct effect (−0.696) and indirect effect (0.036) have opposite signs; H4b was accepted. Within a mediation model, a suppression effect would be present when an indirect effect has a sign that is opposite to that of the total effect (MacKinnon et al., 2000). Therefore, passive leisure participation has a noticeable dampening influence on the effect of smartphone use to reduce levels of depressive symptoms. Smartphone use and mobile Internet access have a positive effect on passive leisure participation, and passive leisure participation has a positive impact on depression symptoms among the older adults, which is consistent with prior work of Wion et al. (2021).

## 5. Discussion

This study investigates the relationship between smartphone use and depressive symptoms among older adults in China using various complementary methods based on data from the 2018 China Longitudinal Aging Social Survey (CLASS). We conduct a comprehensive empirical analysis, beginning with a benchmark linear regression to examine the effect of smartphone use on depressive symptoms. We then perform robustness tests using the PSM method and conduct sensitivity analyses to assess the reliability of our results. Additionally, we conduct heterogeneity analyses based on control variables such as age, gender, and household characteristics. Finally, we explore the mediating role of social participation variables on the effect of smartphone use on depression symptoms. The results are as follows:

First, older adults who reported higher levels of smartphone use showed fewer depressive symptoms. The findings presented above are consistent with the results of several recent studies (Keane et al., 2013; Chang and Im, 2014). The possible reason may be that the information and resources on the smartphone are more abundant, which helps older adults enrich their lives and maintain regular contact with family and friends, expands access to healthcare services, and increases entertainment options or learning opportunities. So the older adults are less prone to depression and show a high level of mental health.

Second, heterogeneity analysis shows that the depressive symptoms of the aged 60 to 69 group are significantly lower than their counterparts for the adults aged 70 years and above when using the smartphone. Regarding subgroups with gender differences, smartphone use has a slightly greater impact on the mental health of female older adults than male older adults. Additionally, the older adults’ depressive symptoms are lower in rural areas than that of the group in urban areas when using a smartphone. The findings presented above appear to be consistent with the results of several recent studies. Zhao and Liu (2020) obtained that the impact of the Internet on the mental health of older adults aged 60 to 69 is significantly higher than the adults aged 70 years and above. Wang (2020) found that smartphone use has a slightly greater impact on the mental health of female older adults than male older adults. Liao et al. (2020) confirmed that phone use for the Internet is significantly associated with lower depression inclination among rural older adults. The possible reason may be partly because the rural older people need the smartphone to communicate with family and friends who have relocated to urban areas for work or study. In addition, rural areas may have limited access to public goods and services such as transportation infrastructure and public entertainment venues, making smartphones and the wealth of information and resources they provide particularly attractive to older adults in these communities.

Third, our analysis also found that political participation, voluntary participation, and active leisure participation serve as mediators between smartphone use and lower levels of depression among older adults in China. Specifically, our results indicate that smartphone use is significantly associated with higher levels of political, voluntary, and active leisure participation among the older adults. This could be attributed to the fact that smartphones provide a medium for older adults to access information about these activities, which may further encourage their participation (Räsänen and Kouvo, 2007). Moreover, we found that political participation, voluntary participation, and active leisure participation have a significant negative impact on depression symptoms in the older adults. The findings are in line with the results of several recent studies. Previous studies confirmed that political participation (Guo et al., 2018), voluntary participation (Wahrendorf et al., 2008), and active leisure participation (Noguchi et al., 2022) significantly reduced the depressive symptoms of the older adults in China. In contrast, passive leisure participation appears to play a suppressing role, and it leads the total effect to appear small. This was consistent with the research findings of Adjei et al. (2017), who believed that passive leisure activities, including listening to the radio or tapes, watching television, and engaging in relaxation, were negatively associated with the mental health of the older adults. Overall, our findings suggest that smartphone use can have positive effects on older adults’ mental health by facilitating their participation in various activities and promoting social engagement.

Based on the above empirical analysis results, this study provides suggestions on how to reduce the severity of depression of the older adults through the use of smartphones, which are as follows: (1) Improve the attitude of older adults toward smartphone use. Given that information and communication technology is thought to be more challenging for the older adults to learn (Hussain et al., 2018), they may fail to accept the convenience brought by the smartphone. Therefore, targeted training services can be provided to enhance the ability of older adults to use various applications on smartphones, such as health management, leisure, and social contact. This can bridge the digital divide and achieve active aging. (2) The government should help create an elderly friendly environment by building activity centers and increasing public service fiscal expenditures. Additionally, social resources can be mobilized for investment in the elderly care industry. (3) The local community should organize various forms of social activities among the aged and publicize them through the smartphones to stimulate their interest in political participation, voluntary participation, and active leisure participation.

This study makes important contributions to the research on the relationship between smartphone use and depressive symptoms among older adults in China. Firstly, it fills the gap in the literature by exploring the role of smartphone use in promoting the psychological health of older adults, which has received relatively little attention compared to Internet use. Secondly, it demonstrates the mediating role of social participation in the relationship between smartphone use and depressive symptoms, which has not been extensively examined in previous studies. Thirdly, it employs robustness tests to ensure the reliability and validity of the findings. Overall, the study provides important insights into how smartphone use can be leveraged to improve the mental health of older adults in China, and can inform the development of policies and interventions to promote active aging.

## 6. Conclusion

This study contributes to the understanding of the complex relationship between smartphone use and depressive symptoms in older adults, and highlights the importance of social participation as a mediator in this relationship. The findings suggest that promoting smartphone use among older adults and encouraging them to engage in social activities such as politics, volunteering, and active leisure can be effective strategies for reducing depression symptoms in this population. However, the study also emphasizes the importance of considering the different types of social participation, as not all forms may have the same impact on mental health. Overall, this study provides valuable insights for policymakers, healthcare providers, and researchers who are interested in improving the mental health and wellbeing of older adults in the context of digital technology.

## Data availability statement

The original contributions presented in this study are included in the article/Supplementary material, further inquiries can be directed to the corresponding author.

## Author contributions

W-CC conceptualized the manuscript and designed the methodology. W-CC and RJ contributed to undertaking the statistical analysis. W-CC wrote the manuscript, with some edits from all authors. All authors read and approved the final manuscript.
