# Supplementary material for: The contribution of the smartphone use to reducing depressive symptoms of Chinese older adults: The mediating effect of social participation
Source: Front Aging Neurosci. 2023 Apr 6;15:1132871. doi: 10.3389/fnagi.2023.1132871 (PMC10117680; doi:10.3389/fnagi.2023.1132871)
Supplement: Supplementary file 1 [file Table_1.docx]

**Table S 1.** Results of balance test

| **Variable** |  | **Mean** | | **Deviation rate(%)** | **t-test** | |
| --- | --- | --- | --- | --- | --- | --- |
|  | **Sample** | **Treatment group** | **Control group** |  | **t-value** | **p>\|t\|** |
| Gender | Unmatched | 0.516 | 0.498 | 3.600 | 1.18 | 0.236 |
|  | Matched | 0.518 | 0.515 | 0.700 | 0.19 | 0.985 |
| Age | Unmatched | 67.206 | 72.149 | -78.400 | -24.14 | 0.000 |
|  | Matched | 67.274 | 67.536 | -4.100 | -1.38 | 0.200 |
| Marital status | Unmatched | 0.836 | 0.667 | 39.800 | 12.40 | 0.000 |
|  | Matched | 0.834 | 0.830 | 0.900 | 0.29 | 0.775 |
| Household | Unmatched | 0.810 | 0.414 | 89.100 | 27.83 | 0.000 |
|  | Matched | 0.808 | 0.806 | 0.500 | 0.15 | 0.924 |
| Education | Unmatched | 1.202 | 0.706 | 90.900 | 29.22 | 0.000 |
|  | Matched | 1.194 | 1.226 | -5.900 | -1.64 | 0.110 |
| Work status | Unmatched | 0.154 | 0.287 | -32.400 | -10.12 | 0.000 |
|  | Matched | 0.154 | 0.166 | -3.000 | -0.90 | 0.528 |
| Housing ownership | Unmatched | 1.163 | 0.989 | 49.100 | 17.16 | 0.000 |
|  | Matched | 1.157 | 1.167 | -2.600 | -0.62 | 0.743 |
| Family income | Unmatched | 10.240 | 9.140 | 97.100 | 30.02 | 0.000 |
|  | Matched | 10.229 | 10.233 | -0.400 | -0.12 | 0.928 |
| Number of children | Unmatched | 1.876 | 2.735 | -72.700 | -22.58 | 0.000 |
|  | Matched | 1.886 | 1.923 | -3.200 | -1.03 | 0.396 |
| ADL score | Unmatched | 21.792 | 21.518 | 19.800 | 5.90 | 0.000 |
|  | Matched | 21.790 | 21.761 | 2.100 | 0.76 | 0.599 |
| IADL score | Unmatched | 17.666 | 17.145 | 29.100 | 8.48 | 0.000 |
|  | Matched | 17.662 | 17.653 | 0.500 | 0.20 | 0.768 |
| Number of comorbid chronic disease | Unmatched | 1.723 | 1.785 | -3.900 | -1.28 | 0.202 |
|  | Matched | 1.716 | 1.742 | -1.600 | -0.46 | 0.931 |

*The results in the table are obtained by using the radius matching method.*

**Table S 2.** The average treatment effect of smartphone use on depression symptoms.

| **Matching method** | **Treatment group**  **(1)** | **Control group**  **(2)** | **ATT value**  **(1)－(2)** | **Standard deviation** | **t-value** |
| --- | --- | --- | --- | --- | --- |
| Before the match ATT | 14.442 | 16.263 | -1.821 | 0.089 | -20.27*** |
| After the match ATT |  |  |  |  |  |
| Radius matching | 14.434 | 15.865 | -1.431 | 0.156 | -9.15*** |
| Caliper nearest neighbor matching | 14.452 | 15.835 | -1.382 | 0.160 | -8.59*** |
| Kernel matching | 14.434 | 15.441 | -1.357 | 0.142 | -9.50*** |

*Note: *p < 0.1, **p < 0.05, ***p < 0.01.*

**Table S 3.** Sensitivity analysis.

|  | Significance levels | | Hodges-Lehmann point  estimates | | 95% Confidence  intervals | |
| --- | --- | --- | --- | --- | --- | --- |
| Gamma  (Γ) | Minimum | Maximum | Minimum | Maximum | Minimum | Maximum |
| 1 | 0 | 0 | -1.4259 | -1.4259 | -1.6066 | -1.2458 |
| 1.2 | 0 | 0 | -1.7061 | -1.1468 | -1.8871 | -0.9658 |
| 1.4 | 0 | <0.0001 | -1.9416 | -0.9123 | -2.1250 | -0.7307 |
| 1.6 | 0 | <0.0001 | -2.1458 | -0.7106 | -2.3333 | -0.5277 |
| 1.8 | 0 | <0.0001 | -2.3250 | -0.5357 | -2.5105 | -0.3487 |
| 2 | 0 | <0.0001 | -2.4834 | -0.3784 | -2.6710 | -0.1916 |
| 2.2 | 0 | 0.0072 | -2.6247 | -0.2388 | -2.8142 | -0.0482 |
| 2.4 | 0 | 0.1271 | -2.7503 | -0.1111 | -2.9439 | 0.0822 |
| 2.6 | 0 | 0.5225 | -2.8684 | 0.0047 | -3.0606 | 0.2005 |

*The results in the table are obtained by using the radius matching method.*

**Data availability statement**

Data were obtained from the National Survey Research Center at the Renmin University of China and are available at http://class.ruc.edu.cn/ (accessed on 12 May 2022) with the permission of the National Survey Research Center at Renmin University of China.
